# Cross-Border Cooperation for One Health in Central Asia: Strengthening Systems and Securing Futures through Regional Health Diplomacy

**DOI:** 10.34172/hpp.45299

**Published:** 2026-06-06

**Authors:** Vijay Kumar Chattu, Nidhi Nagabhatla, Alma Nurtazina, Philippe De Lombaerde, Altay Dyussupov

**Affiliations:** ^1^Department of Public Health, Health Administration & Information and Health Sciences, College of Health Sciences, Tennessee State University, Nashville, TN 37203, USA; ^2^ReSTORE lab, Department of OS & OT, Temerty Faculty of Medicine, University of Toronto, Toronto, ON M5G 1V7, Canada; ^3^Central Asian Regional Central for Planetary Health (CARCPH), Semey Medical University, Semey, 071400, Kazakhstan; ^4^United Nations University Institute on Comparative Regional Integration Studies (UNU-CRIS), Potterierei 72, 8000 Bruges, Belgium; ^5^Department of Economics, Ghent University, Ghent, Belgium; ^6^Brussels School of Governance, Vrije Universiteit Brussel, Belgium

**Keywords:** One Health, Central Asia, Regional cooperation, Regional security, Health diplomacy, Sustainable development

## Abstract

Central Asia faces mounting challenges from emerging infectious diseases, antimicrobial resistance, and food insecurity. This article examines regional cooperation in the One Health approach as a transformative strategy for strengthening regional health security through Regional Health Diplomacy (RHD). The Central Asia One Health Framework for Action represents a paradigmatic shift from fragmented national health strategies to an integrated regional approach. We argue that ‘RHD’ serves as the critical catalyst for implementing the One Health Framework. We call for the strategic implications to extend beyond health outcomes to encompass broader regional integration, establishing Central Asia as a pioneer in multi-sectoral health governance and a potential knowledge hub for One Health implementation.

## Introduction

 In today’s interconnected world, challenges such as emerging and re-emerging infectious diseases, antimicrobial resistance, and food insecurity represent complex threats that cross national borders and require coordinated responses. The World Organization for Animal Health (WOAH) estimates that nearly 75% of new human infectious diseases originate from animals, underscoring the need for integrated human, animal, and environmental health systems at the core of the One Health paradigm.^1^ The Central Asian Region (CAR)—comprising Kazakhstan, Kyrgyzstan, Tajikistan, Turkmenistan, and Uzbekistan—occupies a pivotal geopolitical crossroads linking Europe, Asia, and the Middle East. This vast landlocked area of roughly 4 million square kilometers functions as both a bridge and buffer among major global powers such as Russia, China, Iran, and Afghanistan, making it a strategically significant space in international relations.

**Figure 1:**
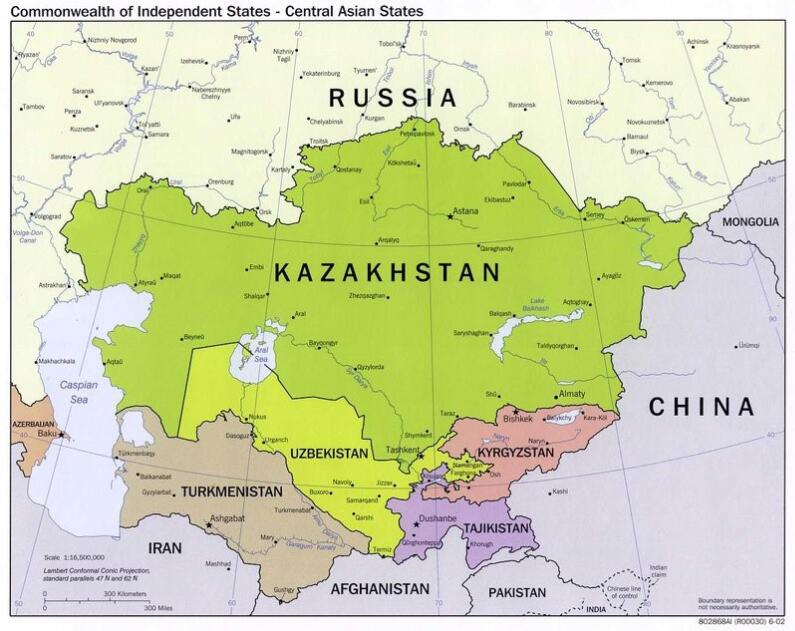
Map of Central Asian Regions showing the five member states (**Source:** UNRCCA, https://unrcca.unmissions.org/map)^3^

 With a population exceeding 83 million in 2025, the region (Figure 1) has experienced remarkable demographic growth, expanding nearly fivefold since 1950 and projected to reach 114 million by 2050. Uzbekistan leads with over 36 million inhabitants, followed by Kazakhstan (20.6 million), Tajikistan (10.6 million), Turkmenistan (7.5 million), and Kyrgyzstan (7.2 million). The population is also notably young, with a median age of 26.7 years, creating both opportunities for economic vitality and challenges in employment, infrastructure, and governance. The region has over 20% of the earth’s uranium reserves, 17% of oil, 7% of natural gas reserves, and considerable concentrations of rare earth elements.^2,3^

 The World Bank’s Regional Director for Central Asia highlighted that the rise of these infections is connected to human intervention with animals’ natural habitats, agricultural techniques that deplete water supplies and biodiversity, and intense farming of a few domesticated animal types.^4^ Also, Central Asian leaders have increasingly positioned health as a national and regional priority. Engagement in multilateral forums and global initiatives reflects recognition that health security is not only a humanitarian concern but also a pillar of sovereignty, competitiveness, and influence in a multipolar world.^5^ The region’s shared ecosystems, agricultural systems, trade routes, and migration patterns create common vulnerabilities but also significant opportunities for cooperation (Table 1).

**Table 1:** Unique features of the Central Asian Region

**S. No**	**Domain**	**Shared values and interests**
1	Geopolitical	The region's strategic location and resources attract the attention of major global powers like Russia, China, European Union and the United States, leading to competition and influence-seeking.^6^
2	Power	The region’s political system shows a strong emphasis on presidential power and limited political opposition. While nominally democratic, these regimes often exhibit characteristics of personalist rule, where power is concentrated in the hands of a single leader.^7^
3	Historical	The countries in the region are all part of the former Soviet Union and the legacy continues to shape political and social structures including the legacy of a centralized, planned economy and the imposition of national borders.
4	Trade	The region is located strategically at the intersection of global value/supply chains that rely heavily on labour migration.^4^
5	Governance	Governance is characterized by authoritarian models.^8^
6	Cooperation	Kazakhstan, Kyrgyzstan and Uzbekistan marked the beginning of regional integration by establishing the Central Asian Union in 1994, which was joined by Tajikistan in 1998. Other intergovernmental organizations have been established by more powerful external actors to promote regional cohesion, such as the Commonwealth of Independent States (CIS), the Eurasian Economic Community (EurAsEC), the Eurasian Economic Union (EEU), and the Shanghai Cooperation Organization (SCO).^9^ Also of relevance is the Organization of Turkic States.
7	Partnerships	Central Asian republics have taken notable steps toward deepening their mutual regional engagement reflecting a maturing political will among Central Asian leaders to define their strategic space, collectively manage regional challenges, and assert their agency in an increasingly multipolar world.^10^
8	Economical	The region's economy has grown steadily, averaging 6.2 percent each year, more than doubling the global average of 2.6 percent in the last decade. Intraregional trade has grown by 4.5 times to $11 billion (from $2.4 billion). Foreign direct investment inflows have nearly doubled from 2016 ($27 billion) reaching $50 billion in 2023.^2^
9	Epidemiological	The zoonotic diseases such as brucellosis, anthrax and Lumpy Skin diseases are common in the region.
10	Animal Health	The region shares animal species, agricultural systems, and trade and movement patterns

 The prioritization of health in the development and political agenda of Central Asian Countries is demonstrated by the active involvement of national leaders or high-ranking officials in several global, regional, and subregional events. These discussions frequently highlight health as a primary priority in the subregion.^5^ The convergence of youthful demographics, strategic geography, and growing political commitment positions Central Asia as a potential laboratory for innovative, integrated health governance approaches. In this perspective, we can argue that *One Health strategies can help the region strengthen its resilience and contribute to addressing global health challenges in the 21st century.*

## Regional Health Security Threats in the Region

 The growing complexity of health threats—ranging from antimicrobial resistance and zoonotic disease outbreaks to food safety risks and climate-driven health shocks—demands a robust One Health strategy. This integrated approach is no longer optional; it is essential to averting future health crises, reducing economic vulnerabilities, strengthening global health security, and advancing sustainable development cost-effectively and holistically.^11^

 As emphasized by the World Bank’s Regional Director for Central Asia, climate change is set to intensify these risks. Yet many countries remain reliant on short-term solutions, particularly the overuse of antimicrobials—antibiotics, antivirals, and antifungals—to manage infectious disease, safeguard food systems, and stimulate animal production. Such practices accelerate one of humanity’s gravest challenges: antimicrobial resistance (AMR), where pathogens evolve to evade treatment, undermining decades of progress in medicine and public health.^4^

 In response, the Central Asia Roadmap for Health and Well-being (CARM) underscores the region’s collective achievements in sustaining political commitment to health despite persistent pressures. From the cascading impacts of climate change and the lingering effects of the COVID-19 pandemic to heightened political instability, Central Asian nations continue to demonstrate resilience and collaboration.^12^ This momentum must now be strategically leveraged to embed One Health at the core of regional policy frameworks—transforming vulnerability into leadership in the global health arena.

###  What is One Health?

 Human activities are the main driving forces behind new, emerging, and re-emerging diseases, and most of the pathogens that are infectious to humans spread from animals. Therefore, managing these global health risks necessitates the full collaboration of the livestock, environmental, and public health sectors at the local, national, regional, and global levels. Recognizing this, the quadripartite agencies, namely the Food and Agriculture Organization (FAO), the World Health Organization (WHO), the United Nations Environment Programme (UNEP), and the WOAH signed an agreement to strengthen the cooperation between nations and optimize the health of humans, animals, plants, and the environment.^13^

**Figure 2:**
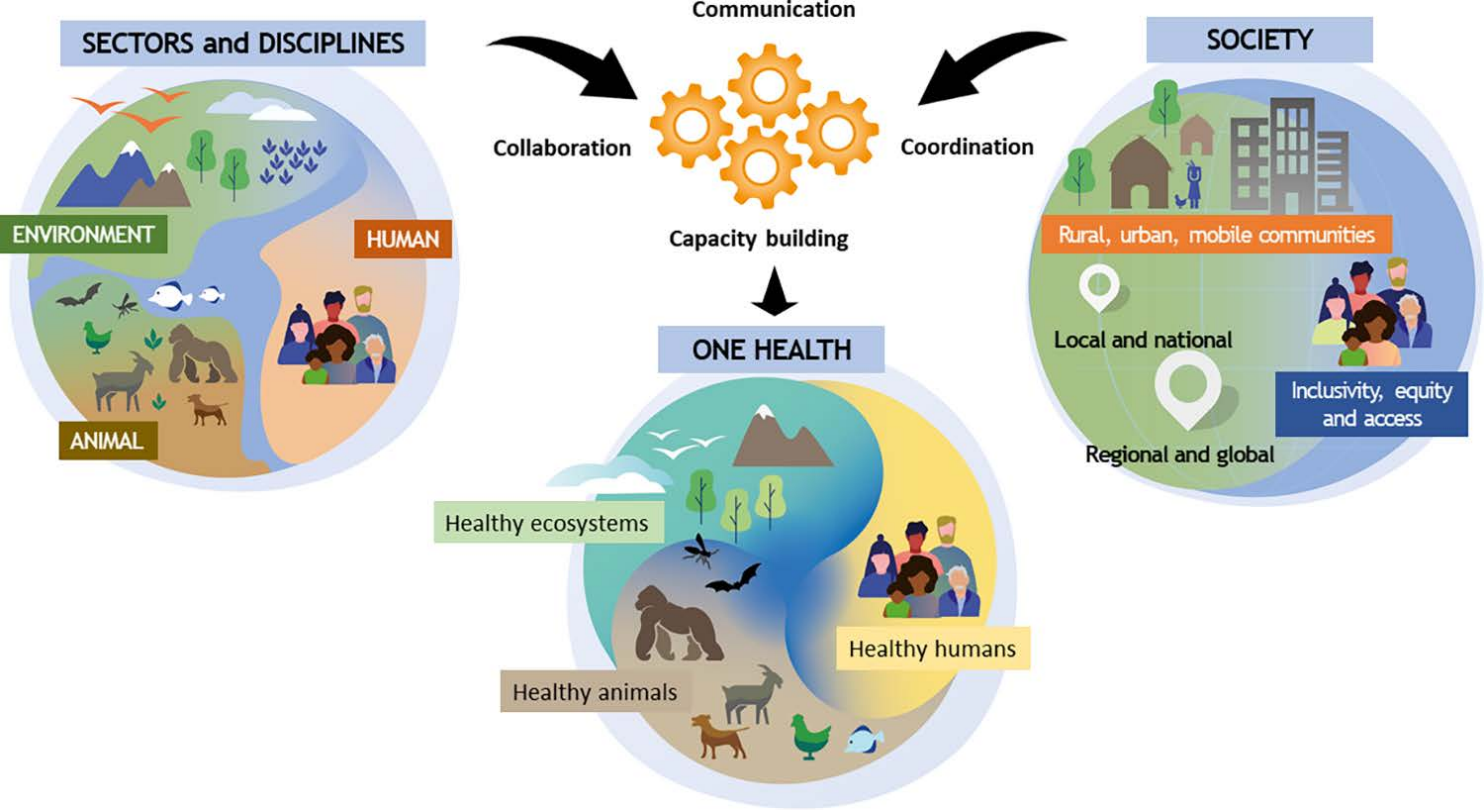
Schematic representation of implementing One Health using a multi-sectoral, multi-level and interdisciplinary approach through 4 ‘C’s: Communication, Coordination, Collaboration, and Capacity-building (**Source**: World Health Organization)^15^

 The One Health High-Level Expert Panel (OHHLEP) defined “One Health as an integrated, unifying approach that aims to sustainably balance and optimize the health of people, animals, and ecosystems. It recognizes that the health of humans, domestic and wild animals, plants, and the wider environment (including ecosystems) is closely linked and interdependent. The approach mobilizes multiple sectors, disciplines, and communities at varying levels of society to work together to foster well-being and tackle threats to health and ecosystems, while addressing the collective need for clean water, energy, and air, safe and nutritious food, taking action on climate change, and contributing to sustainable development”.^14^ The One Health Agreement signed by the quadripartite agencies advocates for implementation through effective planning, communication, collaboration, coordination, capacity building (4C’s), and response efforts through multi-sectoral, multi-level, and interdisciplinary approaches for these complex health challenges (Figure 2). These quadripartite agencies emphasized cooperation to address growing antimicrobial resistance (AMR), emerging and endemic zoonotic diseases (foodborne AMR and diseases), food safety, environmental determinants of health, and health systems strengthening.^15^ The range of issues addressed by this One Health strategy is summarized below (Table 2).

**Table 2:** Range of issues addressed through the One Health Strategy

**S. No**	**Issue/ Area**	**Main reason**
1	Antimicrobial Resistance (AMR)	Bacteria and parasites develop the ability to defeat the drugs designed to kill them and continue growing and spreading;
2	Vector-borne Diseases	People are affected who get bitten by a vector (e.g. mosquitoes, ticks, lice, and fleas), such as dengue fever, West Nile virus, Lyme disease, and malaria;
3	Zoonotic Diseases	Pathogens spread between animals and humans, such as anthrax, brucellosis, avian influenza, and rabies;
4	Food Safety and Foodborne Diseases	Contamination of food at any stage of the food production, delivery, and consumption chain, such as norovirus, salmonella, campylobacter, and listeria;
5	Environment / Climate Change	Anthropogenic global stressors, including land use change, biodiversity loss, climate change and the environmental pollution of soil, water and air, which in turn increase risks to human health;
6	Health Systems	Underperforming functioning of health systems in the prevention and management of infectious zoonotic diseases, production-related diseases, AMR, food safety, and other hazards;
7	Dialogue and Communication between Nations	Need to support regional dialogue between networks of decision makers and technical staff (including epidemiologists, veterinarians, and environmental specialists) from the three operational sectors.
8	Global Health Security	Cases of transboundary disease outbreaks require the sharing of information, the quick integration of new knowledge, and regional action, important building block for pandemic prevention, preparedness, and response;
9	Investment for Humanity’s Future/ Global Solidarity	Need for governments and development partners to avoid the cycle of panic and neglect and direct financial resources. This integrated, risk-based approach requires compliance with international health standards and promotes country ownership, while recognizing its global public goods nature;
10	Sustainable Development	Need for efforts to achieve equity, stewardship, and sustainable development.

**Source: **prepared by the authors.

###  Why One Health is a Unique Opportunity for Central Asia?

 Central Asia’s geographic position at the intersection of global value chains and its reliance on extensive labor migration render the region particularly vulnerable to the emergence and spread of new infectious diseases. Shared characteristics—including common animal species, agricultural systems, migratory routes, and trade networks—create both risks and opportunities, but also provide a strong foundation for developing a coordinated regional response.^4^

 Strategic investment in One Health will deliver wide-ranging co-benefits that extend beyond health security. These include strengthened food safety, biodiversity conservation, and measurable reductions in greenhouse gas emissions, underscoring the integrated value of a One Health approach, as emphasized by Martien van Nieuwkoop, the World Bank’s Global Director for Agriculture and Food.^4^ Recognizing this strategic imperative, the governments of Central Asia formally launched the Central Asia One Health Framework for Action in Almaty on November 14, 2022. This landmark initiative brought together senior representatives from health, agriculture, and environment ministries across the region, signaling a unified commitment to advancing One Health as a cornerstone of regional stability, resilience, and sustainable development.^16^

## Regional Health Governance in the Region

 The WOAH stressed that “[e]ffective global health governance requires strong national capacities along with international coordination”.^17^ The COVID-19 pandemic also illustrated the role and importance of regional cooperation in health. It is crucial for the horizontal and vertical coordination of health policies, the strengthening of disease surveillance, the facilitation and promotion of trade and production of medicines, the joint procurement of medicines, etc.^18^ This calls for a stronger commitment and cooperation among the five member countries of Central Asia. The Director General of WOAH emphasized strengthening global cooperation, ensuring access to safe medicines and vaccines, and taking appropriate control measures.^17^ However, the CAR leadership has succeeded in persuading all the member states to act diligently and commit to the agreed Framework for Action, thereby “setting an example for other regions and countries on how to work together for future generations’ health and wellbeing”.^4^ The CARM has implemented a governance framework to ensure national ownership along with consensus in decision-making. High-ranking representatives, appointed by ministers, promote cooperation within the CARM High-level Standing Group. The Standing Group, endorsed by all Central Asian health ministers, convenes periodically and serves as an institutionalized catalyst for health collaboration within the subregion. It was conceived to be country-centric, is closely overseen by health ministers, and seeks to institutionalize regular ministerial meetings, as evidenced by the establishment of the consultative platform of the Regional Forum – the Meeting of Ministers of Health of CACs. The ratification of the CARM 2022–2024 was emphasized in the Fourth Consultative Meeting of Central Asian Heads of State, convened in July 2022 in Cholpon-Ata, Kyrgyzstan.^19^

 The Framework for Action will support regional dialogue between networks of decision-makers and technical staff, including epidemiologists, veterinarians, and environmental specialists from the three operational sectors. This will be especially useful in cases of transboundary disease outbreaks, as it would enable the sharing of information, the quick integration of new knowledge, and regional action. The summary of various global One Health policies and declarations is summarized below (Table 3).

**Table 3:** List of Global One Health Policies and Declarations in recent years

**Year**	**Declaration/ Policy**	**Purpose**
May 2025	WHO Pandemic Agreement	First international treaty to formally integrate the One Health approach into pandemic prevention, preparedness, and response efforts. It encourages joint training programs for professionals across sectors to build capacities in line with One Health principles
Nov 2024	G20 Health Summit Brazil	Two declarations - the Rio de Janeiro Declaration of the G20 Health Ministers and a G20 Health Ministerial Declaration on Climate Change, Health, and Equity and One Health - were endorsed, representing significant progress toward the global integration of a One Health approach. They urge a comprehensive adoption of One Health strategies to combat complex global health threats, especially those amplified by climate change.
October 2024	G7 Health Ministers’ Communique	The Communique emphasizes the importance of the One Health approach in addressing complex global health challenges - antimicrobial resistance (AMR) and pandemic preparedness, intensified by the impacts of climate change. It calls for continued support to the Pandemic Fund, including expanding the donor base, with support from new sovereign donors, philanthropies, and the private sector. It supports the work of the Quadripartite (FAO, UNEP, WHO, WOAH), to prevent, prepare, and respond to future health emergencies and to promote health through the One Health Approach and the implementation of the One Health Joint Plan of Action
September 2023	G20 New Delhi Leaders’ Declaration	It elevates the position of One Health, underscoring its importance in uniting global health and sustainability. It highlights a global commitment to strengthen health systems and preparedness, with a key focus on the One Health approach.
October 2023	G7 shared understanding of One Health approach	It outlines action tracks that countries can take to implement a One Health approach: Strengthening One Health capacities to strengthen health systems; Reducing the risks from emerging and re-emerging zoonotic epidemics and pandemics; Controlling and eliminating endemic zoonotic, neglected tropical, and vector-borne diseases; Strengthening the assessment, management, and communication of food safety risks; Curbing the silent pandemic of AMR: Integrating the environment into One Health.
September 2023	UNGA 78: Political Declaration of the United Nations General Assembly High-level Meeting on Pandemic Prevention, Preparedness and Response	The Declaration recognizes the interconnectedness of human, animal, and environmental health and acknowledges the need for a coordinated approach across sectors to address health threats. It highlights the importance of strengthening surveillance systems for zoonotic diseases, which can jump from animals to humans. It emphasizes the need for financing mechanisms that support One Health initiatives, particularly in low- and middle-income countries.
March 2022	Quadripartite collaboration on One Health	Four international agencies – FAO, the United Nations Environment Programme (UNEP), the World Health Organization (WHO), and the World Organisation for Animal Health (WOAH), signed a groundbreaking agreement: to strengthen cooperation to sustainably balance and optimize the health of humans, animals, plants and the environment. advocating for One Health implementation at international events, such as G20 Meetings, the United Nations Climate Change Conference, and the World One Health Congress.

**Source: **prepared by the authors.

 Besides, the regional membershave signed key environmental multilateral initiatives such as: 1) United Nations Framework Convention on Climate Change (UNFCCC), 2) Convention on Biological Diversity (CBD), 3) the United Nations Convention to Combat Desertification (UNCCD), 4) the Ramsar Convention, 5) the Stockholm Convention on Persistent Organic Pollutants, and 6) the Vienna Convention for the Ozone Layer, which includes the Montreal Protocol.^20^

## Regional Health Diplomacy for One Health in the Central Asian Region

 Regional Health Diplomacy has only recently received scholarly attention.^21^ It can refer to inter-state negotiations in the field of health or related to health, it can refer to the impact on health of negotiations in other areas (e.g. trade), and/or it can refer to negotiations leading to forms of regional health governance. In the latter sense, it is centered on leveraging health-related concerns to promote collaboration and enhance health outcomes in a particular region. Addressing common health issues and advancing regional stability requires the involvement of multiple players, including governments, international organizations, and civil society.^22^ RHD brings together stakeholders, ministerial departments, and disciplines such as public health, international law, international affairs, economics, information technology, and management to shape regional health policy and promote health and peace through effective negotiations.^23^ The active involvement of regional member states in global health decision-making is vital to safeguarding their strategic interests and ensuring their voices shape the international health agenda. Central Asia must strengthen its regional presence in global dialogues that address critical threats such as antimicrobial resistance, epidemics, and bioterrorism, as well as the broader determinants of health linked to climate change, lifestyle transitions, and commercial influences—including tobacco marketing and the taxation of sugar-sweetened and high-salt foods. To implement the One Health Framework for Action (OHFA) effectively, regional stakeholders—spanning human, animal, plant, and environmental health, as well as the foreign policy, finance, and human resources sectors—must recognize the need for deeper engagement and sustained collaboration.

 The World Organization for Animal Health^17^ emphasizes that advancing a data-driven approach to One Health requires coordinated action among the three core domains of human, animal, and environmental health. Strengthening data-collection systems, promoting transparency, and enhancing mechanisms for global data-sharing are essential. Achieving these priorities calls for strong communication, coordination, and collective action at both national and regional levels. By embedding these practices within RHD, the region can ensure the seamless implementation of the One Health agenda, positioning Central Asia as a proactive and credible contributor to global health security and sustainable development (Table 4).

**Table 4:** Regional One Health activities in Central Asia and the role of Regional Health Diplomacy

July 2025	Regional Environmental Centre for Central Asia (CAREC) and the World Bank Meeting in Uzbekistan	“One Health for Pandemic Prevention, Food System Resilience, and Ecosystem Health in Central Asia”. The One Health project focuses on the interconnectedness of human, animal, and environmental health, aiming to prevent pandemics, build resilient food systems, protect ecosystems, and foster cross-sectoral collaboration for sustainable development.
Feb 2025	National Coordination Meeting under the project “Pandemic Preparedness and Response through One Health approach in Central Asia” in Turkmenistan	To improve health, economic stability, social advancement, and environmental sustainability in the region. Adopting a One Health approach to pandemic prevention, preparedness, and response (PPR), they are working together with the support of the Quadripartite Alliance for One Health.
Feb 2024	14th Meeting of the Conference of the Parties to the Convention on the Conservation of Migratory Species of Wild Animals (CMS COP14) on “One Health in Central Asia,” in Samarkand, Uzbekistan.	Seeks to address the relationship between environmental, animal, and human health. Promotes a comprehensive approach to solving complicated problems.
May 2023	ASEAN Leaders’ Declaration on One Health Initiative	Emphasizes collaboration and coordination across sectors – human health, animal health, and plant health – as well as with environmental agencies. Acknowledges the need for a multi-stakeholder approach, involving governments, non-governmental organizations, and international partners.
November 2022	Central Asia One Health Framework For Action	Aims to contribute to addressing three high-level goals shared among Central Asian countries: pandemic prevention and preparedness, resilience of food systems, and improving regional trade and the competitiveness of agriculture. Identify focus areas and mechanisms for regional collaboration, and include a One Health dashboard to monitor progress, while facilitating policy responses to emerging issues.
July 2022	One Health Meeting in Tashkent	Agreed on the need to prepare the Central Asia One Health Framework for Action provide a blueprint for the countries in the region to move forward with concrete actions, as well as include a roadmap for investments at national and regional levels.

###  RHD Activities for the Effective Implementation of the Framework for Action

 As a cross-sectoral approach, One Health can be most effectively advanced through RHD, providing a strong platform for negotiating shared priorities and preventing future pandemics. Mittal et al., in their bibliometric analysis on global health diplomacy, have highlighted how health diplomacy can connect international borders and promote cooperation through effective governance among nations with shared interests.^24^ Therefore, RHD provides the strategic vehicle to achieve this, reinforcing cross-sectoral cooperation while embedding health security within broader agendas of regional stability, socioeconomic growth, and national resilience. The One Health Framework for Action (OHFA), emerging from successful RHD among regional partners, is designed to deliver on three critical goals: strengthening pandemic prevention and preparedness, building resilient food systems, and enhancing regional trade and agricultural competitiveness. Through RHD, the OHFA can establish clear policy responses to emerging concerns, identify priority areas for collaboration, and create dedicated channels for regional engagement. A central feature will be the One Health Dashboard, which will enable partners to monitor progress, share data, and strengthen coordination. This mechanism will not only improve communication among regional stakeholders but also foster collaboration between networks of technical experts and decision-makers, including environmental specialists, veterinarians, and epidemiologists across the three core sectors. We also point out that by facilitating real-time information exchange, rapid integration of new knowledge, and coordinated regional action, the OHFA will provide a vital platform for responding to transboundary disease outbreaks. In doing so, it strengthens collective resilience and positions the region to respond more effectively to the evolving global health landscape.^4^ Using RHD as a health promotion strategy improves regional collaboration across health, foreign policy, and other sectors. In addition to strengthening ties between countries, RHD promotes regional growth and efficient governance to meet global goals.^25^

###  Way Forward

 Looking forward, the Central Asian One Health initiative serves as a critical test case for whether regional integration can effectively address 21st-century global challenges. The success of this framework will likely influence similar efforts in other regions facing comparable threats, making Central Asia a potential knowledge hub for One Health implementation in developing contexts. The emphasis on pandemic prevention, food security, and environmental sustainability positions the region strategically within global discourse on climate adaptation and health system strengthening, potentially unlocking increased development finance and technical cooperation. However, the long-term sustainability of this initiative will depend on continued political commitment across leadership transitions, sustained financing mechanisms, and the ability to adapt the framework to emerging health threats and geopolitical shifts while maintaining the collaborative spirit that enabled its creation.

## Conclusion

 The Central Asian cooperation in One Health represents a paradigmatic shift from fragmented national health strategies to an integrated regional approach that leverages shared geopolitical, historical, and epidemiological vulnerabilities as strengths for collective resilience. We reflect on how Regional Health Diplomacy can function as the critical catalyst for operationalizing the One Health Framework for Action, transforming the theoretical underpinnings of the quadripartite alliance (FAO, WHO, UNEP, WOAH) into concrete regional mechanisms for pandemic prevention, food systems resilience, and enhanced agricultural competitiveness. The strategic significance lies not merely in adopting One Health principles, but in the innovative use of Central Asia’s unique characteristics—shared animal species, common agricultural systems, established cooperative frameworks, and interconnected migration patterns—as the foundation for a replicable model of regional health security.

 The Framework for Action, launched in November 2022, represents more than a policy document; it embodies a diplomatic achievement that positions Central Asia as a pioneer in multi-sectoral health governance. Through systematic implementation of the 4 C’s framework (Communication, Coordination, Collaboration, and Capacity-building), the region has created sustainable institutional mechanisms, including the CARM High-level Standing Group and regular ministerial consultations, that ensure national ownership while maintaining regional coherence. This governance architecture addresses the fundamental challenge of balancing sovereignty concerns with collective action imperatives, particularly crucial given the region’s historical experience with external influence and its current navigation of multipolar geopolitical pressures.

 In addition, strategic implications extend beyond health outcomes to encompass broader regional integration and global positioning. By successfully mobilizing RHD to address complex challenges ranging from antimicrobial resistance to climate-related health threats, Central Asian countries have demonstrated their capacity for autonomous problem-solving and regional leadership. This has significant ramifications for their engagement with major powers and international organizations, as it establishes credibility as a unified regional bloc capable of implementing sophisticated policy frameworks. The One Health dashboard and data-sharing mechanisms further enhance this strategic positioning by creating transparency and accountability systems that build trust among regional partners while demonstrating progress to international stakeholders.

## Competing Interests

 Vijay Kumar Chattu is an editorial board member of Health Promotion Perspectives. All the other authors declare no competing interests in this work.

## Data Availability Statement

 Data is available from the corresponding author upon request.

## Ethical Approval

 Not applicable.
